# Consideration in Platelet Transfusion-Transmitted Klebsiella pneumoniae Sepsis: A Case Report

**DOI:** 10.7759/cureus.109140

**Published:** 2026-05-18

**Authors:** Arthur V.D. Rezende, Livia Dahmen Rodrigues, Maria del Pilar Bayona Molano, Arman Dagal, Tiago Macruz

**Affiliations:** 1 Medicine, Federal University of Pelotas, Pelotas, BRA; 2 Anesthesiology, University of Miami, Miami, USA; 3 Interventional Radiology, University of Miami, Miami, USA

**Keywords:** bacterial infections, klebsiella pneumoniae, platelet transfusion, portasystemic shunt, septic shock, transfusion-transmitted infection

## Abstract

Transfusion-transmitted bacterial infection (TTBI) is most commonly associated with platelet transfusions. Although multiple preventive measures are routinely implemented, the risk of bacterial contamination during blood donation, processing, or storage cannot be completely eliminated. In this context, Gram-negative bacteria such as *Klebsiella pneumoniae* may lead to fulminant sepsis, particularly in immunocompromised patients, including those with underlying liver disease.

We describe the case of a 57-year-old man classified as American Society of Anesthesiologists (ASA) III who underwent a transjugular intrahepatic portosystemic shunt (TIPS) procedure. Due to preoperative thrombocytopenia, platelet transfusion was initiated. However, approximately 15 minutes after the start of the transfusion, the patient developed tachycardia and oxygen desaturation intraoperatively, which, despite supportive measures, rapidly progressed to septic shock and multiorgan failure, ultimately resulting in death. Blood cultures grew *K. pneumoniae*, while bronchoalveolar lavage cultures were negative. Molecular analysis later confirmed that a second patient who received platelets from the same donor developed an infection caused by an identical bacterial strain, establishing transfusion-transmitted bacterial contamination.

Although similar cases have previously been reported in the literature, this case reinforces the importance of robust hemovigilance systems, continuous improvement of transfusion safety protocols, and early recognition of septic transfusion. It also highlights the need for heightened awareness among healthcare professionals, particularly anesthesiologists, regarding signs of transfusion-associated sepsis during the perioperative period.

## Introduction

Transfusion-transmitted bacterial infection (TTBI) is a major infectious complication of blood products and is most frequently associated with platelet transfusions [1]. Unlike red blood cells (RBCs) or plasma, platelets must be stored at 20°C-24°C with continuous agitation. These conditions, combined with gas-permeable bags, create an environment that allows even small bacterial inocula to multiply rapidly. In any patient in whom fever develops within six hours after platelet infusion, the possibility of bacterial contamination of the component should be examined [2,3]. However, recent surveillance data indicate that septic transfusion reactions may also appear later, between nine and 24 hours after transfusion, highlighting the need for ongoing clinical vigilance beyond the immediate post-infusion period [4].

Despite improvements in donor screening, culture testing, and pathogen reduction, cases of septic transfusion episodes continue to occur, often with severe or fatal outcomes [1,5]. The estimated risk of TTBI from platelets is one in 50 thousand to one in 100 thousand, far higher than the risk of transfusion-transmitted viral infections [1]. Gram-positive skin flora are the most common contaminants, but Gram-negative organisms such as *Klebsiella pneumoniae* can cause fulminant sepsis, being more deadly than Gram-positive bacteria [3,6]. Furthermore, certain populations have heightened susceptibility to TTBI due to immune dysfunction [6], particularly cirrhotic patients.

To highlight the specific risks for these vulnerable cohorts, we report a fatal case of *K. pneumoniae* sepsis following platelet transfusion in a patient with cirrhosis undergoing transjugular intrahepatic portosystemic shunt (TIPS). Evidence of transfusion transmission was supported by the occurrence of the same strain in another recipient from the same donor. Written informed consent for the publication of this case report was obtained from the patient’s legal representative.

## Case presentation

A 57-year-old male patient with alcohol-associated cirrhosis, hepatic encephalopathy, esophageal variceal hemorrhage, partial superior mesenteric vein thrombosis, and hematemesis was classified as American Society of Anesthesiologists (ASA) III and scheduled for a TIPS procedure. Preoperative laboratory evaluation revealed a platelet count of 41,000/µL (Table 1). Due to thrombocytopenia, he received one unit of single-donor platelet transfusion preoperatively.

**Table 1:** Parameters recorded throughout the preoperative, immediate postoperative, first day postoperative, and second day postoperative aPTT: activated partial thromboplastin time; HCO₃⁻: bicarbonate; INR: international normalized ratio; NR: not recorded; pCO_2_: partial pressure of carbon dioxide; pO₂: partial pressure of oxygen; RBC: red blood cell; WBC: white blood cell

Parameter	Normal value	Preoperative	Immediate postoperative	First day postoperative	Second day postoperative
Platelets	150,000-450,000/µL	41,000/µL	33,000/µL	51,000/µL	75,000/µL
RBC count	4.7-6.1 million cells/µL (male)	3.46 million cells/µL	3.69 million cells/µL	1.96 million cells/µL	4.67 million cells/µL
WBC count	4,500-11,000/mm	2,600/mm	2,400/mm	5,300/mm	32,300/mm
INR	0.8-1.2	1.35	15.10	3.37	4.62
Fibrinogen	200-400 mg/dL	182 mg/dL	<35 mg/dL	56 mg/dL	81 mg/dL
aPTT	25-40 seconds	34 seconds	>200 seconds	NR	97 seconds
Arterial pH	7.35-7.45	NR	7.31	7.18	7.11
Arterial PCO_2_	35-45 mmHg	NR	33 mmHg	30 mmHg	38 mmHg
Arterial PO_2_	80-100 mmHg	NR	111 mmHg	93 mmHg	82 mmHg
Arterial HCO_3_^-^	22-26 mEq/L	NR	16 mEq/L	11 mEq/L	12 mEq/L
Arterial base excess	-2-+2 mmol/L	NR	-10 mmol/L	-17 mmol/L	-18 mmol/L
O_2_ Sat arterial	95%-100%	NR	97.3%	95.3%	95.4%
Lactate arterial	0.5-2.2 mmol/L	1.8 mmol/L	5.8 mmol/L	14.8 mmol/L	15.1 mmol/L

The patient underwent TIPS under general anesthesia. Intraoperatively, approximately 15 minutes after the initiation of transfusion, the patient developed acute tachycardia and oxygen desaturation to an SpO₂ of 80%, without fever or hypotension. This immediate temporal association strongly suggested a transfusion-related event. Postoperative laboratory testing obtained two hours later revealed profound coagulopathy, with international normalized ratio (INR) > 15.1, activated partial thromboplastin time (aPTT) > 200 s, and fibrinogen < 35 mg/dL (Table 1).

Within seven hours, the patient's respiratory status required endotracheal intubation. Concurrently, he developed refractory septic shock, requiring infusions of norepinephrine, vasopressin, methylene blue, hydrocortisone, and continuous infusion of epinephrine, in addition to mechanical ventilation and renal replacement therapy. Blood cultures were obtained, and empiric broad-spectrum antibiotics with vancomycin and piperacillin-tazobactam were initiated.

**Figure 1:**
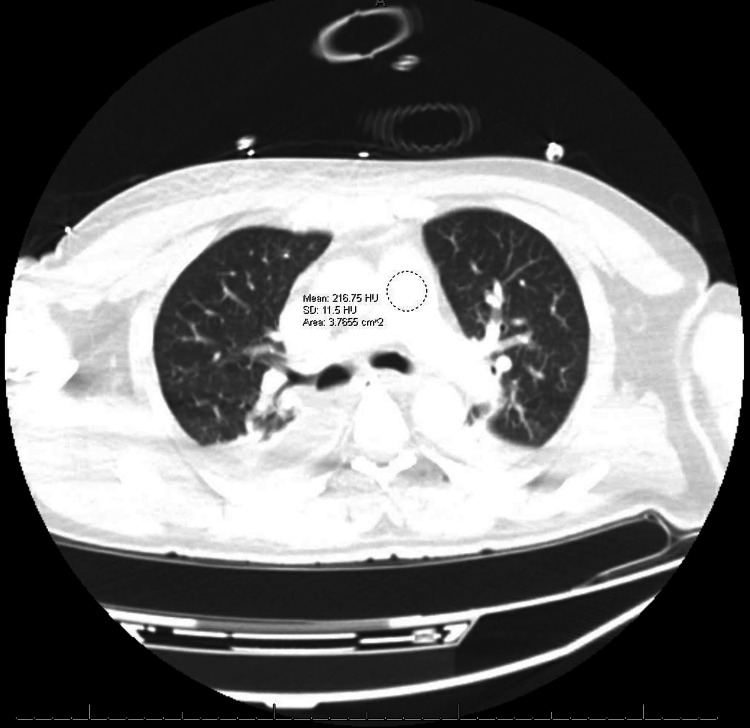
CT scan performed one day postoperatively demonstrating bilateral infiltrates

Despite an initially normal postoperative chest radiograph, a CT scan performed 12 hours after the procedure showed bilateral infiltrates (Figure 1). Fourteen hours after the procedure, the patient developed progressive hypoxemia, requiring mechanical ventilation with escalating settings. PaO₂/FiO₂ ratios ranged between 100 and 200, dropping to consistently <100 by the following day. Peak pressures reached 37-40 cm H₂O, with a positive end-expiratory pressure (PEEP) of 12 cm H₂O and FiO₂ requirements of 70%-100%, findings consistent with severe acute respiratory distress syndrome (ARDS).

Over the second and third postoperative days, the patient required massive transfusion support, including 11 units of RBCs, six units of thawed pooled fibrin (TPF), five units of thawed plasma (PLA), five units of fresh frozen plasma, and one unit of platelets on the second day. And subsequently, on the third postoperative day, he was administered two units of TPF, two units of plasma, and one unit of platelets. Hemostatic parameters began to improve only after multiple blood component transfusions. Blood cultures obtained on the day of the procedure grew *K. pneumoniae*, while bronchoalveolar lavage cultures remained negative.

Metabolic dysfunction was also pronounced. Arterial blood gases demonstrated severe lactic acidosis, with lactate rising from 5.8 mmol/L during the procedure to 14.8 mmol/L the next day (Table 1). Despite intensive support, the patient progressed to multiorgan failure and died three days after the surgical intervention.

Notably, for hemovigilance purposes, all transfusion units were tracked by unique identification numbers. It is important to highlight that all blood components underwent standard screening procedures, including bacterial contamination testing. Specifically regarding platelet units, bacterial screening was performed according to institutional protocols.

A transfusion service investigation revealed that another patient who received platelets from the same donor developed sepsis due to *K. pneumoniae* with an identical antibiogram (Table 2). Molecular diagnostic testing confirmed that both patients’ post-transfusion blood cultures grew the same bacterial strain, establishing the causal link between the two infections.

**Table 2:** Antibiogram of blood culture with resistance profile of the bacteria Klebsiella pneumoniae of patients who received platelet transfusion

	Index patient	Secondary recipient
Klebsiella pneumoniae		
Ampicillin	Resistant	Resistant
Ampicillin/sulbactam	Susceptible	Susceptible
Cefazolin (nonurine)	Susceptible	Susceptible
Cefazolin (urine)	Susceptible	Susceptible
Cefoxitin	Susceptible	Susceptible
Ceftazidime	Susceptible	Susceptible
Cefuroxime	Susceptible	Susceptible
Gentamicin	Susceptible	Susceptible
Levofloxacin	Susceptible	Susceptible
Meropenem	Susceptible	Susceptible
Piperacillin/tazobactam	Susceptible	Susceptible
Trimethoprim/Sulfa	Susceptible	Susceptible

The implicated platelet component itself was not cultured, as it had already been discarded by the time the transfusion was completed. However, the donor’s co-component, a unit of plasma obtained from the same apheresis donation, was cultured by the blood center and showed no bacterial growth. The platelet component had been collected exclusively by apheresis, and no other blood components from the same donor were transfused into the index patient. The time between donor apheresis and patient transfusion was five days.

## Discussion

During the procedure, the patient developed sudden tachycardia and oxygen desaturation shortly after platelet transfusion. In the absence of hypotension or fever, these findings were initially nonspecific and could be attributed to several perioperative causes. Although underlying cirrhosis and the procedure itself may have contributed to clinical instability, the marked laboratory abnormalities were highly suggestive of disseminated intravascular coagulation (DIC), reinforcing the suspicion of transfusion-related sepsis. This presentation highlights an important diagnostic challenge in anesthetic practice: transfusion-transmitted sepsis may initially manifest with subtle physiologic alterations that overlap with more common intraoperative events, such as allergic reactions, ventilatory issues, or hemodynamic fluctuations related to anesthesia.

For anesthesiologists, continuous interpretation of trends rather than isolated vital sign abnormalities is critical. Any unexplained clinical deterioration temporally associated with blood component administration should prompt consideration of septic transfusion reaction and should not be prematurely dismissed in the absence of classic infectious signs. Under general anesthesia, fever, chills, and rigors are frequently absent or delayed, increasing the importance of vigilance for early markers such as tachycardia, hypoxemia, metabolic acidosis, or coagulopathy.

Early recognition has direct therapeutic implications. A recent systematic review demonstrated that initiation of antimicrobial therapy within three hours of symptom onset significantly reduces mortality in sepsis, regardless of shock severity [7]. In the present case, the definitive diagnosis was established only after postoperative deterioration, emphasizing how early intraoperative manifestations may represent the first opportunity for intervention. Furthermore, certain patient populations may present increased susceptibility to TTBI due to immune dysfunction [6], particularly cirrhotic patients, such as the patient described in this case.

Importantly, molecular typing confirmed that both the index patient and another recipient of platelets from the same donor were infected with genetically identical *K. pneumoniae*, establishing a transfusion-transmitted event. Although the implicated platelet unit was unavailable for culture, the sterile plasma co-component and trace-back investigation support platelet-specific contamination.

*K. pneumoniae* is part of the normal gastrointestinal microbiota and may also colonize human skin, increasing the possibility of contamination during blood collection or processing [8]. In addition, Gram-negative organisms such as *K. pneumoniae* are frequently associated with more severe transfusion-related septic reactions because of the presence of multiple virulence mechanisms, which may contribute to rapid progression to severe sepsis and shock [8].

A similar fatal transfusion-transmitted *K. pneumoniae* infection was reported by Horth et al. In that report, a patient received an apheresis platelet unit and developed vomiting, tachycardia, and hypotension within 15 minutes of transfusion initiation, progressing to death within five hours. Investigation confirmed contamination by *K. pneumoniae* [9].

The precise source of contamination could not be definitively established. Potential explanations include donor microbiota contamination during collection or contamination related to the blood processing procedure. Additionally, although the clinical and epidemiological findings strongly support transfusion transmission, intestinal bacterial translocation associated with the TIPS procedure cannot be completely excluded. Nevertheless, the temporal relationship between transfusion and symptom onset, the molecular confirmation of identical isolates, epidemiological linkage between recipients, and the literature reinforce the transfusion-related hypothesis.

A major limitation of this case was the absence of a positive culture from the implicated platelet unit, which prevented direct microbiological confirmation of the blood component itself. Furthermore, the patient received additional blood transfusions during subsequent hospitalization. However, considering the chronology and clinical evolution, these later transfusions were not associated with new reactions or clinical worsening suggestive of additional transfusion-related events. Although current evidence suggests that earlier antimicrobial administration improves survival in sepsis, it cannot be definitively concluded that earlier antimicrobial therapy would have altered the outcome in the present case.

While adherence to transfusion safety protocols is high, cluster investigations and surveillance studies have demonstrated that bacterial contamination of platelets may still occur despite donor screening, bacterial testing, and pathogen reduction strategies [9-15]. Residual risk persists even with preventive measures such as skin disinfection, diversion of the initial blood collection volume, bacterial cultures, and pathogen inactivation technologies [16].

Currently, several pathogen reduction and contamination prevention strategies are available for platelet transfusions, including riboflavin-based technologies, amotosalen, ultraviolet light, bacterial detection assays, and structured hemovigilance systems [14,16-18]. In the institution where this event occurred, transfusion safety measures are implemented in collaboration with OneBlood and include donor screening, skin disinfection, diversion of the initial blood collection volume, bacterial testing, pathogen inactivation technologies, and active hemovigilance protocols. However, even within robust safety systems, rare events may still occur. This highlights that patient safety depends not only on system-level safeguards but also on continuous clinical awareness and rapid multidisciplinary response.

This case also emphasizes the ongoing need for improvement in transfusion safety technologies, including the development of next-generation pathogen reduction systems and rapid detection assays capable of identifying bacterial contamination earlier and more reliably. Recent literature emphasizes that prevention of transfusion-transmitted infection depends not only on blood bank protocols but also on frontline clinical recognition [15-17]. From an anesthesiology perspective, this case reinforces that vigilance during transfusion must be considered an active and continuous process. Anesthesiologists are uniquely positioned to recognize early physiologic deterioration, initiate prompt investigation, and communicate concerns to transfusion medicine and hemovigilance services while supportive measures and antimicrobial therapy are considered.

## Conclusions

Given the severity of the clinical presentation and fatal outcome, this case highlights the importance of hemovigilance systems and reinforces the need for healthcare professionals to remain attentive to early signs of transfusion-related sepsis. It also emphasizes the importance of a judicious, evidence-based approach to transfusion therapy and reinforces the anesthesiologist's central role in perioperative patient safety. Maintaining a high index of suspicion for septic transfusion reactions during unexplained intraoperative deterioration may allow earlier recognition and intervention, even in the absence of classic infectious signs.

Finally, this report reinforces several important clinical lessons. Early recognition of transfusion-related sepsis may improve patient outcomes by enabling prompt interruption of transfusion, rapid collection of blood cultures, initiation of broad-spectrum antimicrobial therapy, activation of hemovigilance protocols, and immediate communication with transfusion services. Rapid recognition may also prevent additional adverse outcomes by facilitating timely investigation of potentially contaminated blood products and reducing the risk of exposure of additional recipients to infected donor components.
